# Unveiling the Secret Chemistry of Street Art by a Multitechnique Approach

**DOI:** 10.1002/cplu.202500059

**Published:** 2025-05-06

**Authors:** Elena C. L. Rigante, Francesca Modugno, Jacopo La Nasa, Silvia Pizzimenti, Tommaso R. I. Cataldi, Cosima D. Calvano

**Affiliations:** ^1^ Department of Chemistry University of Bari Aldo Moro via Orabona 4 70126 Bari Italy; ^2^ Department of Chemistry and Industrial Chemistry University of Pisa 56121 Pisa Italy; ^3^ Centro Interdipartimentale SMART University of Bari Aldo Moro via Orabona 4 70126 Bari Italy

**Keywords:** binders, mass spectrometry, pigments, polymers, pyrolysis–gas chromatography/mass spectrometry, street art

## Abstract

In recent years, graffiti and street art have gained recognition as legitimate art forms, deserving of the same care and attention as traditional art. As a result, conservators and restorers are now working to develop standardized guidelines for the cleaning, conservation, and restoration of these vibrant works. Our study takes a closer look at the materials used in street art, specifically the spray varnishes used by artists. Samples from two murals created in 2021 in Bari, Italy, are analyzed using a range of advanced techniques such as attenuated total reflection Fourier‐transform infrared spectroscopy, reversed‐phase liquid chromatography coupled with UV‐Vis and electrospray ionization mass spectrometry (MS), and laser desorption ionization MS as well as pyrolysis–gas chromatography/MS. Acrylic, polyvinyl acetate, and styrene–acrylic resins are identified as the primary binders used in street art spray varnishes, along with common additives such as polyethylene and polypropylene glycol. The organic dyes and pigments, such as yellow (PY74), orange (PO36), red (rhodamine), and blue (phthalocyanine) hues used to create colorful images of street art, are also characterized. This study demonstrates the importance of a multitechnique approach in understanding the complex chemistry of modern spray varnishes used in street art.

## Graphical abstract

Two murals created in 2021 in Bari, Italy, have been analyzed by attenuated total reflection Fourier‐transform infrared spectroscopy, reversed‐phase liquid chromatography coupled with UV‐Vis and electrospray ionization mass spectrometry (MS), laser desorption ionization MS, pyrolysis–gas chromatography/MS. Acrylic, polyvinyl acetate, styrene–acrylic resins as primary binders along with additives such as polyethylene and polypropylene glycol were identified in spray varnishes.

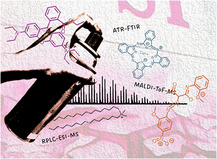

## 1 Introduction

Graffiti, which originated as an act of rebellion and frustration in ethnic neighborhoods of the United States, was initially dismissed as mere vandalism.^[^ ^1^ ^]^ Over time, however, graffiti and murals, once viewed as part of deviant subcultures,^[^ ^2^ ^]^ have gained recognition as legitimate forms of art. Despite their cultural value, they remain illegal in many countries unless specifically authorized. Recently, conservators and art historians have shown a growing interest in preserving these artworks, recognizing their vulnerability to vandalism and environmental factors. This has spurred efforts among scientists and restorers to develop standardized conservation guidelines, restoration strategies, and chemical analysis protocols.^[^ ^3^ ^]^ Significant progress has been made in understanding spray paint composition^[^ ^4^, ^5^, ^6^, ^7^, ^8^ ^]^ and its aging process,^[^ ^9^ ^]^ which is essential for designing effective cleaning and preservation methods.^[^ ^10^, ^11^, ^12^ ^]^ Spray varnishes are complex formulations containing pigments, dyes, polymers, propellants, fillers, and various additives, including drying agents, surfactants, plasticizers, and emulsifiers. Since these formulations are often proprietary and not disclosed on labels, it is crucial to conduct thorough diagnostic campaigns to identify their components.^[^ ^7^, ^13^ ^]^ Organic or inorganic pigments are usually solvent‐dispersed in a polymeric acrylic, styrenic, or alkyd‐based binder. Very important roles are fulfilled by additives which strongly affect the varnish properties and stability to ageing and can be drying agents, thickeners, surfactants, antiskinning agents, plasticizers, antiflooding agents, dispersants, and emulsifiers.^[^ ^14^, ^15^, ^16^ ^]^

The complex formulation of modern spray varnishes necessitates a comprehensive analytical strategy that combines multiple complementary techniques. Fourier‐transform infrared (FTIR) spectroscopy serves as an essential first step, providing rapid identification of inorganic pigments and enabling differentiation between binder types such as acrylic and alkyd resins through their characteristic vibrational group.^[^ ^5^, ^6^, ^7^, ^17^, ^18^, ^19^, ^20^, ^21^, ^22^ ^]^ Building on this foundation, pyrolysis–gas chromatography/mass spectrometry (Py‐GC/MS) offers deeper molecular insights, capable of identifying specific polymers through their thermal degradation products while simultaneously detecting additives like plasticizers and fillers.^[^ ^5^, ^7^, ^22^, ^23^ ^]^ For the analysis of organic colorants and polymeric additives, advanced mass spectrometry (MS) techniques prove particularly valuable. Electrospray ionization (ESI) and matrix‐assisted laser desorption/ionization (MALDI) MS provide sensitive detection of dyes and medium molecular weight additives (1000–2000 Da), with each ionization method offering unique advantages for different compound classes.^[^ ^24^, ^25^ ^]^ The integration of reversed‐phase liquid chromatography with diode array detection and MS (RPLC‐DAD‐ESI‐MS) creates a particularly powerful platform for dye characterization, combining separation efficiency with both spectroscopic and mass spectrometric identification. For insoluble pigments such as phthalocyanines that challenge chromatographic analysis, laser desorption ionization (LDI) MS emerges as an effective solution, enabling direct analysis without matrix interference.

This multitechnique approach has demonstrated its effectiveness in numerous real‐world applications. A recent 2024 investigation of David Alfaro Siqueiros’ “Untitled Mural 3” used μ‐FTIR, SEM‐EDS, and high‐resolution NMR to fully characterize the artwork's complex material system, identifying acrylic and polyvinyl acetate binders, titanium white, and iron oxide pigments, along with various fillers and plasticizers.^[^ ^26^ ^]^ Similarly, the examination of Jorit Agoch's “Ama Il Tuo Sogno” combined polarized light microscopy, Py‐GC‐HRAMS, and SEM‐EDS to not only determine the chemical composition of paints and mortar supports but also to diagnose humidity‐related salt damage.^[^ ^27^ ^]^ The fading mechanisms in the vibrant fluorescent hues of Pontevia and Castagnetti's “UBUNTU” were successfully elucidated through an innovative combination of μ‐Raman, surface‐enhanced Raman spectroscopy (SERS), and hyphenated chromatography‐MS techniques.^[^ ^25^ ^]^

In our current study of nine commercial acrylic‐based spray varnishes, this integrated analytical philosophy guides our methodology. We begin with attenuated total reflectance (ATR)‐FTIR for initial compositional screening, followed by Py‐GC/MS for detailed polymer characterization. MALDI‐MS targets medium molecular weight additives, while reversed‐phase liquid chromatography coupled with diode array detection and ESI‐MS (RPLC‐DAD‐ESI‐MS) provides a comprehensive analysis of soluble dyes through correlated UV‐Vis and mass spectral data. For challenging insoluble components such as phthalocyanines, LDI‐MS or MALDI‐MS offers a direct analysis pathway; LDI‐MS guarantees rapid detection without matrix interference. This tiered approach ensures complete material characterization while overcoming the inherent limitations of any single technique, providing conservation scientists with the detailed information needed to develop appropriate preservation strategies for complex artistic materials. This multitechnique approach was also applied to murals in the Quartiere Museale San Paolo in Bari, Italy, created by the artists Hogre and Manu Invisible. The analysis revealed that the spray varnishes used in these artworks contained styrene and styrene‐acetate resins as primary binders, with polyethylene and polypropylene glycol identified as additives through MALDI‐MS. Organic dyes and pigments were characterized using LDI‐MS(/MS) and HPLC‐DAD‐ESI‐MS and tandem MS, allowing the identification of yellow (PY74), orange (PO36), red (rhodamine), and blue (phthalocyanine) pigments and dyes.

The primary objective of this study is to advance the scientific understanding of contemporary spray varnishes used in street art through comprehensive material characterization. Our work contributes new insights into the field of graffiti and mural conservation by 1) developing optimized analytical protocols for complex, multilayered spray paints; 2) identifying both declared and undeclared components in commercial products; and 3) establishing correlations between laboratory reference samples and real‐world artworks. These findings directly support the development of improved conservation strategies, including compatible protective coatings,^[^ ^28^, ^29^ ^]^ and more effective cleaning methods for vandalism removal.^[^ ^30^, ^31^ ^]^ By integrating these insights, our work aligns with ongoing efforts to enhance the preservation of urban and cultural heritage.

## 2 Experimental Section

### 2.1 Chemicals

Water LC‐MS grade, methanol, acetone, dimethyl sulfoxide (DMSO), chloroform, 2‐propanol (IPA), *trans*‐2‐[3‐(4‐tert‐butylphenyl)‐2‐methyl‐2‐propenylidene]malononitrile (DCTB), dithranol, NaTFA, NaI, renin, angiotensin I, ACTH, and nylon syringe filters 13 mm 0.20 μm pore size were purchased from Sigma Aldrich (Milan, Italy).

### 2.2 Varnishes and Mural Samples

**Figure 1:**
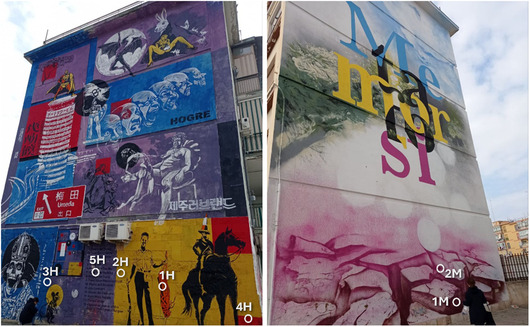
Two of the ten murals realized for the project “Quartiere Museale San Paolo”. *Wikipedia Unusual Articles*, Hogre, 2021 on the left, and *Metamorfosi*, Manu Invisible, 2021 on the right. Sampled points are highlighted.

Nine commercial acrylic spray varnishes from different brands and colors, as listed in **Table** **1**, were analyzed. These varnishes are versatile and can be applied to various surfaces such as wood, plastics, stone, and glass, both for indoor and outdoor use. To identify any minor differences in their chemical composition, varnishes with the same binder type (acrylic resin, as specified on the varnish label) but from different brands were selected. Here is how the samples were prepared: a) each spray varnish was applied onto microscope glass slides or aluminum slides and b) allowed to dry for 1 week at room temperature (≈21 °C). Real varnish samples (each ≤ 10 mg) were collected from murals at the Quartiere Museale San Paolo (QM San Paolo) in Bari, Italy. Two specific artworks were considered: *Wikipedia Unusual Articles* by Hogre (2021) and *Metamorfosi* by Manu Invisible (2021). **Figure** **1** illustrates the two murals with the sampled points marked, which are also detailed in **Table** **2** along with their corresponding colors and codes. For the field samples collected from actual murals, our sampling strategy involved obtaining one specimen for each colored varnish present in the artwork. This process presented significant practical challenges due to the large scale of the murals, which limited our access primarily to the lower sections of each work. The size constraints meant we could only safely collect samples from areas within reach while still ensuring representative sampling across the different colored varnishes used in each mural. The sample pretreatment required for each analysis is described in the following sections. Notably, no pretreatment was necessary for ATR‐FTIR experiments.

**Table 1:** List of acrylic varnish sprays examined by multitechnique strategy from different brands and color.

#	Branda)	Color
1	Arexonsb)	Yellow
2	Cilvanic)	Yellow
3	Molotow Flame oranged)	Yellow
4	Molotow Flame orange	Magenta
5	Molotow Flame orange	Blue
6	Molotow	Blue fluo
7	Molotow	Pink fluo
8	Capece)	Yellow
9	Veloxf)	Blue

a) Varnishes can be applied to various surfaces such as wood, plastics, stone, and glass, both for indoor and outdoor use.

b) AREXONS S.p.A, https://arexons.it.

c) Cilvani SRL, https://cilvani.it/.

d) MOLOTOW^TM^, https://www.molotow.com/.

e) Liantonio vernici SRL, www.liantoniovernici.com.

f) Cia Technima Sud Europa SRL.

**Table 2:** Specific points sampled on the murals shown in Figure 1.

Mural	Sample	Color
*Wikipedia Unusual Articles* (Hogre)	1H	Orange
	2H	Yellow
	3H	Blue
	4H	Red
	5H	Violet
*Metamorfosi* (Manu Invisible)	1M	Bordeaux
	2M	Pink

### 2.3 ATR‐FTIR

Analysis with attenuated total reflectance FTIR spectroscopy was carried out with the Spectrum Two FTIR Spectrometer (PerkinElmer Inc.) equipped with a diamond prism, directly placing the varnished slides onto the prism. All spectra were acquired by collecting 16 scans in transmission mode in the range 4000–400 cm^−1^ with 2 cm^−1^ resolution. At least two points were analyzed for each sample. The background was registered against air. Spectra interpretation was carried out by comparison with literature data and with OpenSpecy free database (https://osf.io/3uatf/)^[^ ^32^ ^]^ or INFRA‐ART ‐ Spectral Library (https://infraart.inoe.ro/).^[^ ^33^ ^]^ The software Spectrum 10 (PerkinElmer Inc.) was used to acquire and process raw ATR‐FTIR spectra.

### 2.4 Py‐GC/MS

For the Py‐GC/MS analysis, the varnish sample was placed in a quartz tube and heated to 550 °C for 0.2 min. The pyrolyser system used was the CDS Pyroprobe 5000 series, coupled online with a 6890 N GC System Gas Chromatograph and a 5973 Mass Selective Detector single quadrupole mass spectrometer, both from Agilent Technologies, Palo Alto, CA, USA. The pyrolyser interface was kept at 280 °C, the transfer line at 300 °C, and the valve oven at 290 °C. The gas chromatographic separation was performed using an HP‐5MS fused silica capillary column, with a 5% diphenyl‐95% dimethyl‐polysiloxane stationary phase, measuring 30 m in length and 0.25 mm in internal diameter. A deactivated silica precolumn, 2 m in length and 0.32 mm in internal diameter, was also used. The split–splitless injector was used in split mode at 300 °C, with a split ratio of 1:30. The chromatographic conditions were as follows: 40 °C isothermal for 6 min, 20 °C min^−1^ up to 310 °C, and isothermal for 40 min. The carrier gas (He, purity 99.9995%) was used in the constant flow mode at 1.0 mL min^−1^. The observed *m/z* range was 35–420.

### 2.5 MALDI‐ToF‐MS

The analyses were performed with a 5800 MALDI‐ToF/ToF analyzer (AB SCIEX, Darmstadt, Germany) equipped with a neodymium‐doped yttrium lithium fluoride (Nd:YLF) laser (345 nm), in a reflectron positive mode, with 20 ppm mass accuracy. The standard peptide mixture of renin, angiotensin I, and ACTH was used for external mass calibration in the range of 500–6000 *m/z*, which is the range chosen when analyzing polymeric binders in samples. LDI‐MS for dyes and pigments characterization was performed in reflectron mode, negative or positive ionization mode depending on the need, with 20 ppm mass accuracy in the range of 100–1000 *m/z*. Usually, 1000 laser shots were accumulated with a random raster scan at 400 and 1000 Hz laser pulse rates in MS and MS/MS modes, respectively. Each mass spectrum was averaged on at least five acquisitions. The delayed extraction time was set at 400 ns. Two milligrams of varnish was scraped and dissolved in 1 mL of acetone or THF and of DMSO for magenta and red dyes. The solution was ultrasonicated for 30 min at 45 °C. Then, the sample was mixed with matrix DCTB 10 mg mL^−1^ in acetone and dopant NaI 2 mM in IPA:H_2_O 1:1 (v:v) in proportion 10:10:1 (v:v:v, sample:matrix:dopant). 0.5 μL of the mixture was deposited on the target plate in three replicates and dried at room temperature. For pigments/dyes analysis in LDI mode, 0.5 μL of the dissolved sample were directly deposited on the target plate in three replicates. Raw MALDI‐MS(/MS) files were processed using the software Data Explorer 4.11. The software mMass 5.5.0.0. was used for the peak list extraction from txt files.

### 2.6 RPLC‐DAD‐ESI‐MS(/MS)

For chromatographic analysis, we prepared sample solutions at a concentration of 2 mg mL^−1^, which were first ultrasonicated at 45 °C for 30 min to ensure complete dissolution. After this preparation step, identical aliquots of these solutions were used for both MALDI‐ToF‐MS and LC analyses. Before injection into the chromatographic system, the solutions were filtered through 0.45 μm nylon syringe filters. LC‐ESI‐MS analysis was carried out using a Q‐Exactive (Thermo Scientific, Waltham, MA, USA) mass spectrometer equipped with an Orbitrap analyzer and coupled with an Ultimate 3000 Dionex (Thermo Scientific) chromatographer via HESI (heated ESI) interface. An Accucore Polar Premium, 100 × 2.1 mm column (Thermo Scientific, Milano), was used operating at a flow rate of 0.200 mL min^−1^ and *T* = 30 °C. The mobile phase composition was H_2_O (A) and ACN (B) both added with FA 0.1 % v/v.^[^ ^34^ ^]^ The mobile phase gradient used was the following: min 0, B phase 30%, min 2 isocratic B phase 30%, min 7 gradient to 60% B, min 17 gradient to 95% B, min 20 isocratic 95% B, min 22 gradient to 30% B, and min 27 isocratic 30%B. Tandem MS experiments were performed in Data Dependent (DD) mode with a higher‐energy collisional dissociation fragmentation regime at 35% a.u. (arbitrary units) energy. MS parameters were set as the following: 3.5 kV spray voltage, 320 °C capillary temperature, 35 a.u. (arbitrary units) sheet gas flow rate, 15 a.u. of auxiliary gas flow rate, S‐lens radio‐frequency level 60 a.u. Polarity was set as positive or negative depending on the sample. The *m/z* range was 200–2000 while resolution was 70,000 for full‐MS and 17,500 for DD‐MS^2^ scans. DD‐MS^2^ events were performed on the top 5 ions (only mono‐ and doubly charged).

## 3 Results and Discussion

### 3.1 Binder Characterization of Acrylic Spray Varnishes

**Figure 2:**
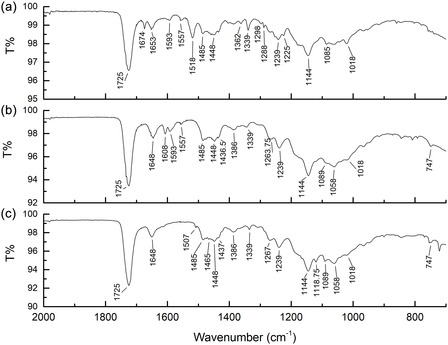
ATR‐FTIR spectra of three Molotow Flame Orange varnishes a) yellow, b) magenta, and c) blue after spray deposition onto glass slides. The main peaks are labeled.

FTIR spectroscopy is a widely recognized tool for obtaining preliminary insights into the binders, fillers, and pigments of varnishes. In this study, we utilized nondestructive ATR‐FTIR spectroscopy to analyze the colored spray varnishes listed in Table 1. As an example, **Figure** **2** presents the ATR‐FTIR spectra of three Molotow varnish: yellow, magenta, and blue. As expected, all three spectra revealed the presence of an acrylic binder,^[^ ^35^ ^]^ with a good agreement (Pearson's *R* = 0.9) to the *n*‐butylacrylate–methyl methacrylate copolymer spectrum from the Open Specy free database.^[^ ^32^ ^]^ Key spectral features included the sharp carbonyl stretching peak at 1725 cm^−^^1^, C—O stretching signals at 1239 cm^−^^1^ and 1144 cm^−^^1^, and characteristic bending vibrations of the polymer backbone between 1485 and747 cm^−^^1^.^[^ ^36^, ^37^, ^38^, ^39^ ^]^ Minor differences in the spectra were attributed to pigment or dye variations. For example, peaks at 1674–1085 cm^−^^1^ in the yellow varnish correspond to arylide yellow (monoazo dye PY74), confirmed by characteristic signals in the 1660–1530 cm^−^^1^ range.^[^ ^38^ ^]^ In the magenta varnish, signals at 1640–1550 cm^−^^1^ and 1390–1340 cm^−^^1^ suggest the presence of quinacridone pigments, specifically PR122.^[^ ^36^, ^37^, ^38^, ^39^ ^]^ Low‐intensity bands at 1507–1058 cm^−^^1^ in the blue varnish point to the presence of a phthalocyanine pigment.^[^ ^40^, ^41^ ^]^

While ATR‐FTIR provides rapid, nondestructive analysis, it has limitations, including signal overlap and sensitivity issues.^[^ ^42^ ^]^ To overcome these, we complemented our findings with Py‐GC/MS, a powerful technique for detailed binder characterization. Chromatograms of the yellow, magenta, and blue varnishes (Figure S1, Supporting Information) confirmed the presence of pyrolysis markers for an acrylic resin based on methyl acrylate and *n*‐butyl acrylate. Additionally, dimethylpalmitamine and dimethylstearylamine, common plasticizers used as additives, were detected.

To further investigate additives, MALDI‐MS analysis was used, which is particularly useful for identifying polymeric compounds like polyethylene glycol (PEG).^[^ ^24^ ^]^ Figure S2, Supporting Information, displays MALDI‐MS spectra of the magenta varnish, revealing a polymeric distribution centered at *m/z* 500 with a 44.0 Da spacing (see asterisked peak signals), corresponding to the ethylene oxide (C_2_H_4_O) monomer. This indicates the presence of PEG, likely added as an emulsifier or humectant acrylic paints.^[^ ^43^, ^44^, ^45^ ^]^ Additionally, minor polymeric distributions with a delta mass of 86.0 Da were observed, likely corresponding to polymethyl acrylate oligomers. Interestingly, *n*‐butyl acrylate, identified in Py‐GC/MS, was not detected in MALDI‐MS spectra, likely due to suppression effects from more polar oligomers. Therefore, the combination of ATR‐FTIR, Py‐GC/MS, and MALDI‐MS provided complementary insights into the chemical composition of the varnishes, enabling the identification of binders, pigments, and additives. This multitechnique approach highlights the complexity of spray varnishes and underscores the importance of using diverse analytical methods to fully characterize such materials.

### 3.2 Pigment/Dye Characterization in Acrylic Varnish Sprays

Pigments and dyes were characterized with greater reliability using MS and chromatography.^[^ ^46^ ^]^ LDI was initially used to investigate pigments and dyes in varnish formulations, with sample solutions (2 mg mL^−1^ in acetone or DMSO) directly spotted onto the target plate without a matrix. This LDI‐MS approach proved to be both convenient and effective, as no matrix‐related interference occurred in the explored mass range (100–1000 *m/z*), and the structures of the pigments and dyes remained well‐preserved after laser energy absorption. As an example, the blue fluorescent varnish from the Molotow brand was analyzed using LDI(+)‐MS. A characteristic isotopic profile with a monoisotopic peak at *m/z* 575.0 was observed (Figure S3A, Supporting Information), corresponding to copper phthalocyanine (PB15), an organometallic pigment with a porphyrin ring structure.^[^ ^47^ ^]^ This widely used blue pigment, commonly found in printing inks, paints, and plastics, is known for its scarce solubility in both polar and organic solvents, which prevented its retrieval during chromatographic analysis.^[^ ^48^ ^]^ Additionally, three signals at *m/z* 494.4, 522.5, and 550.5 were detected, separated by 28 Da, and were frequently identified during the analysis of other varnishes in positive ionization mode.

To gain further structural insights, tandem mass experiments were performed. These three species exhibited a similar fragmentation pattern, exemplified in plot B of Figure S3, Supporting Information, for the precursor ion at *m/z* 494.4. Product ions at *m/z* 43.0, 57.1, 71.1, 85.1, and 99.1 were tentatively assigned to carbocations (C_3_H_7_^+^, C_4_H_9_^+^, C_5_H_1__1_^+^, C_6_H_1__3_^+^, and C_7_H_1__5_^+^), while the peak at *m/z* 46.0 corresponded to C_2_H_8_N^+^. Larger product ions at *m/z* 242.2, 270.3, and 298.3 were attributed to fragments of quaternary ammonium long‐chain salts, as also suggested by pyrolysis products detected via Py‐GC/MS. These fragments were identified as hexadecan‐1‐aminium (*m/z* 242.2), octadecan‐1‐aminium (*m/z* 270.3), and N,N‐dimethyloctadecan‐1‐aminium (*m/z* 298.3), which are generated from quaternary ammonium compounds such as N‐hexadecyl‐N,N‐dimethylhexadecan‐1‐aminium (*m/z* 494.4), N,N‐dimethyl‐N‐octadecyloctadecan‐1‐aminium (*m/z* 550.5), and dioctadecylammonium (*m/z* 522.5). These findings confirmed the role of ammonium long‐chain salts as additives or surfactants in most of the analyzed varnishes.

**Figure 3:**
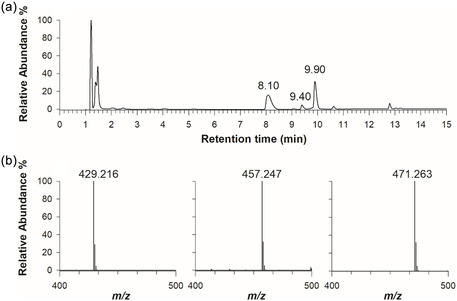
a) PDA chromatogram of the Molotow Pink fluo varnish showing three chromatographic peaks at retention times of 8.10, 9.40 and 9.90 min. b) Positive ion mode FTMS spectra of the three dyes at *m/z* 429.216, 457.247, and 471.263.

To complement LDI‐MS, liquid chromatography coupled with diode array (PPLC‐DAD) and ESI‐MS was used to further characterize these dyes. This approach proved particularly useful for formulations containing dye mixtures or when subtle structural modifications were present. Photodiode array (PDA) chromatograms were instrumental in determining the retention times of colorant species and correlating them with corresponding mass spectrometric data. The high mass accuracy (≤5 ppm) provided by the orbital trap MS instrument allowed for a confident comparison of observed *m/z* values with theoretical masses, surpassing the accuracy of MALDI or LDI data. For example, the pink Molotow fluorescent varnish revealed three UV‐Vis absorbing species at retention times of 8.10, 9.40, and 9.90 min (**Figure** **3**a). The corresponding mass spectra are shown in Figure 3b. These *m/z* values were attributed to modified rhodamines.^[^ ^25^ ^]^ The monoisotopic mass value of conventional rhodamine B and rhodamine 6 G (devoid of their chloride counterion) [M]^+^ is 443.2329 Da, which was absent in the spectra. However, the UV‐Vis spectra associated with the chromatographic peaks (Figure S4, Supporting Information) matched the absorbance profiles of rhodamine 6 G (RT 8.10 min, *λ*_max_ = 529 nm) and rhodamine B (RTs 9.40 and 9.90 min, *λ*_max_ = 560 nm).^[^ ^49^ ^]^ The three dyes were identified as modified rhodamines: a demethylated rhodamine 6 G with a monoisotopic mass of [M]^+^ 429.2173 Da, a methylated rhodamine B ([M]^+^ 457.2486 Da), and an ethylated rhodamine B ([M]^+^ 471.2642 Da), as shown in plot b of Figure 3. Hypothetical derived structures of rhodamine 6 G and rhodamine B, supporting these experimental findings, are illustrated in Figure S5, Supporting Information. To further investigate these structures, MS/MS spectra were examined, though they provided limited information. The precursor ion at *m/z* 429.216 exhibited a loss of both an ethyl radical and neutral ethene (29/28 Da), potentially from oxygen or nitrogen, indicating the proposed structures *1*, *2*, and *3*. For the ion at *m/z* 457.247, a neutral nominal loss of 44 Da (corresponding to a carboxyl residue) suggests that structures *5* and *6* are more plausible. The species at *m/z* 471.263 primarily showed the loss of ethene, supporting structure 7 as the most likely candidate. A comprehensive summary of these findings for the nine varnishes is presented in **Table** **3**.

**Table 3:** Overview of analytical results for all nine brands of varnish films on glass slides.

n.	Varnish branda)	Color	Binders	Pigments/dyes
1	Arexons	Yellow	*n*‐Butylacrylate methylmethacrilate copolymer	PY74, PO36
2	Cilvani	Yellow	Styrene butyl methacrylate polymer	PY74, PO36
3	Molotow Flame orange	Yellow	*n*‐Butylacrylate methylmethacrilate copolymer	PY74
4	Molotow Flame orange	Magenta	*n*‐Butylacrylate methylmethacrilate copolymer	PR122
5	Molotow Flame orange	Blue	*n*‐Butylacrylate methylmethacrilate copolymer	PB15 (copper phtalocyanine)
6	Molotow	Blue fluo	*n*‐Butylacrylate methylmethacrilate copolymer	PB15
7	Molotow	Pink fluo	*n*‐Butylacrylate methylmethacrilate copolymer	Modified rhodamine B, modified Rhodamine 6 G
8	Capec	Yellow	Styrene *n*‐Butylacrylate methylmethacrilate copolymer	PY74
9	Velox	Blue	Acrylic	PB15

a) Polyethylene glycol was identified as additive in all varnishes.

### 3.3 Analysis of Mural Varnish Samples: Insights into Composition, Aging, and Pigments

**Figure 4:**
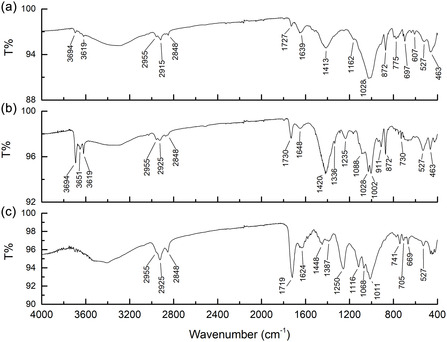
ATR‐FTIR spectra of varnish samples: a) 1H (orange) and b) 3H (blue) from *Wikipedia Unusual Articles*) and c) 1M (bordeaux varnish) from *Matamorfosi*.

Less than 10 mg samples were collected by scraping from different colored areas of the murals *Wikipedia Unusual Articles* and *Metamorfosi*. ATR‐FTIR investigations were performed on these samples, and examples are provided in **Figure** **4**. Unfortunately, very few indications of binders were obtained from ATR‐FTIR spectroscopy. Vibrational bands associated with calcite (broad 1410–1420 cm^−^^1^, sharp 872 cm^−^^1^) and silicates such as kaolinite or gypsum (3694, 3651, 3619, 1028, 1002, and 527 cm^−^^1^) dominated the spectra, occupying the spectral range necessary for confidently identifying binder types. These compounds were possibly related to a fresh plaster layer applied before mural creation as a ground‐preparing layer or served as additives acting as fillers or color modulators in the varnish formulations.^[^ ^17^, ^50^ ^]^ The possibility of a fresh plaster layer is not excluded, as building façades are typically cleaned, smoothened, and treated with primers and preparatory layers before murals are created. Some bands attributed to a polystyrene fraction were identified in sample 1H (2915, 2848, and 697 cm^−^^1^), while other minor IR bands in sample 1M were assigned to a quinacridone dye (1624, 741, 705, and 669 cm^−^^1^).^[^ ^40^, ^41^ ^]^ Additionally, carbonyl stretching at 1720–1730 cm^−^^1^ and CH_2_ stretching in the range of 2850–2955 cm^−^^1^ suggested the use of polyacrylate‐based or polyvinyl acetate binders. A broad band at 3300–3400 cm^−^^1^ was caused by hydroxylated species formed through exposure to atmospheric agents, while a band at 1640–1650 cm^−^^1^ in samples from *Wikipedia Unusual Articles* was attributed to the increased presence of unsaturated species due to aging.^[^ ^51^ ^]^ Previous studies have observed that after both artificial and natural aging, the intensity of IR bands corresponding to binder components tends to decrease.^[^ ^51^ ^]^ Organic polymers are subject to photo‐oxidation and may progressively be removed from the outermost layers by atmospheric agents such as rain.^[^ ^51^ ^]^ Moreover, pigments have been shown to sometimes accelerate binder aging.^[^ ^52^, ^53^ ^]^

**Figure 5:**
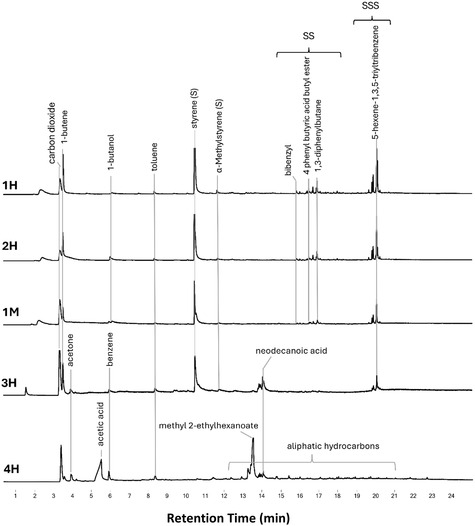
Py‐GC‐MS chromatograms of the samples 1H, 2H, 3H and 4H (orange, yellow, blue, and red varnish, respectively, from *Wikipedia Unusual Articles*) and 1M (Bordeaux varnish from *Matamorfosi*). S: styrene monomer, SS: styrene dimers, SSS: styrene trimers.

The use of Py‐GC/MS was very useful for analyzing real samples. Chromatographic profiles of samples from both murals are shown in **Figure** **5**. The samples from *Metamorfosi* were characterized solely by the presence of polystyrene resin, while those from *Wikipedia Unusual Articles* contained pyrolytic products consistent with polystyrene resin and polyvinyl acetate resin plasticized with an internal plasticizer (VeoVa). Polystyrene and polyvinyl acetate were detected either individually or together, depending on the sample. The binders identified through Py‐GC/MS for each sample are summarized in **Table** **4**. MALDI‐MS in positive ion mode proved effective for the ionization of polar polymers in the analyzed mural samples. As shown in Figure S6, Supporting Information, at least four polymer distributions with a 58 Da monomer mass unit were detectable in three samples. This mass unit was attributed to the monomer of polypropylene glycol (PPG), which was detected in all three samples. A more detailed inspection of the spectrum of sample 1H revealed a less intense distribution of PEG. Both polymers likely functioned as emulsifying additives, consistently with findings in commercial spray varnishes.

**Table 4:** Results from the multianalytical approach on mural samples.

Mural	Sample	Color	Binder	Additives	Pigment, dyes
*Wikipedia Unusual Articles* (Hogre)	1H	Orange	Polystyrene resin	Calcitea), kaolinite, PPG, PEG	PY74, PY154
	2H	Yellow	Polystyrene resin	Calcitea), kaolinite, PPG, PEG	PY154
	3H	Blue	Polystyrene resin, polyvinyl acetate resin, VeoVa^TM^	Calcitea), kaolinite, PEG	PB15
	4H	Red	polyvinyl acetate resin, VeoVa^TM^	Calcitea), kaolinite, PEG	PR254
	5H	Violet	Polystyrene resin	Calcitea)	PB15, PV19
*Metamorfosi* (Manu Invisible)	1M	Bordeaux	Polystyrene resin,	Calcitea), kaolinite	PY74, PR122
	2M	Pink	Polystyrene resin	Calcitea), kaolinite	PR192, PR122

a) Likely coming from the mural support.

LDI‐MS was crucial for identifying copper phthalocyanines as insoluble pigments in samples 3H (blue) and 5H (violet) (Figure S7, Supporting Information). The blue varnish samples were insoluble in all tested solvents, making liquid chromatographic analysis unfeasible. A peak signal at *m/z* 191.0 was identified as the structure proposed in spectrum 3H (Figure S7, Supporting Information), formed through in‐source fragmentation of the porphyrin ring. In the violet varnish sample 5H, the LDI spectrum in positive ion mode revealed copper phthalocyanine along with a mass peak at *m/z* 385.1, attributed to PY74.^[^ ^24^ ^]^ This was likely contamination from the nearby use of orange varnish. Additionally, LDI‐MS revealed other dyes in the varnish formulations of real samples. For instance, the LDI‐MS spectrum in the negative mode for sample 2H (Figure S7, Supporting Information) identified pigment yellow 154 (PY154), a benzimidazolone dye with a theoretical mass of 404.0976 Da in its deprotonated form. This dye is widely used in decorative paints, automotive coatings, plastics, and outdoor products due to its excellent light‐ and weather‐resistance properties.^[^ ^54^ ^]^

**Figure 6:**
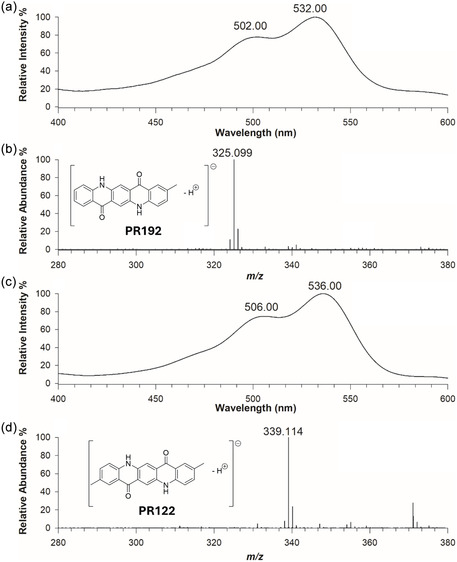
a, c) UV and b, d) HRMS spectra in negative ion mode of dye components from sample 2M (pink, *Metamorfosi*) eluting at retention times of a, b) 13.30 min and c, d) 14.30 min RPLC. In HRMS, these compounds were identified as Pigment Red 192 (b) and Pigment Red 122 d). The molecular structures of the pigments are displayed in the corresponding insets.

RPLC‐DAD‐ESI‐MS also contributed significantly to the investigation of dyestuffs. For sample 2M, two UV‐Vis absorbing compounds were localized in the PDA chromatogram, displaying similar UV‐Vis spectra (**Figure** **6**). The Vis‐absorbance spectra in plots A and C of Figure 6 indicated that the compounds shared structural similarities. Their monoisotopic masses in negative ionization mode ([M‐H]^‐^) were measured as *m/z* 325.099 and 339.114, differing by 14.015 Da, which is the mass of a methylene group (CH_2_). These species were identified as two quinacridone dyes, namely pigment red 192 (PR192) and pigment red 122 (PR122), with monoisotopic masses of 325.0983 and 339.1139 Da, respectively, in their deprotonated forms.

The combined use of these analytical techniques provided a wealth of information about the composition of spray varnishes, which is invaluable for further assessments of aging processes.

## 4 Conclusions

The chemical characterization of real‐world varnish samples posed several challenges, requiring a comprehensive analytical approach. ATR‐FTIR and Py‐GC/MS techniques effectively identified polyacrylate as the primary binder in all commercial samples. However, MALDI‐MS faced limitations in characterizing methacrylate polymers due to its preferential ionization of polar PEG oligomers, commonly present as surfactants, plasticizers, or humectants. In contrast, LDI‐MS proved more suitable for detecting pigments such as copper phthalocyanine and dyes like PY74 and PO36, offering a rapid and direct analytical solution. RPLC‐DAD‐ESI‐MS, combined with tandem ESI‐MS, provided deeper insights, enabling the identification and structural elucidation of key compounds within these varnish formulations. This integrated methodology demonstrated effectiveness in analyzing both laboratory‐prepared and real‐world samples, including varnish samples collected from two murals, revealing crucial information about spray varnish compositions and their interactions. Future research will focus on studying the effects of aging on varnishes applied to both glass slides and authentic mural samples. This continued investigation aims to enhance the understanding of varnish formulation behaviors over time and refine analytical techniques for their evaluation, contributing to advancements in the preservation and conservation of cultural heritage materials.

## Conflict of Interest

The authors declare no conflict of interest.

## Supporting information

Supplementary Material

## Data Availability

The data that support the findings of this study are available from the corresponding author upon reasonable request.
