# Supplementary material for: Unveiling the Secret Chemistry of Street Art by a Multitechnique Approach
Source: Chempluschem. 2025 May 6;90(7):e202500059. doi: 10.1002/cplu.202500059 (PMC12261049; doi:10.1002/cplu.202500059)
Supplement: Supplementary file 1 — Supplementary Material [file CPLU-90-e202500059-s001.pdf]

UNVEILING THE SECRET CHEMISTRY OF STREET ART BY A MULTI-TECHNIQUE APPROACH

Elena C. L. Rigante[1], Francesca Modugno[2], Jacopo La Nasa[2], Silvia Pizzimenti[2], Tommaso R. I. Cataldi[1,3], Cosima D. Calvano[1,3*]

1. Department of Chemistry, University of Bari Aldo Moro, via Orabona 4, 70126 Bari (Italy)

2. Department of Chemistry and Industrial Chemistry, University of Pisa, Pisa

3. Centro Interdipartimentale SMART, University of Bari Aldo Moro, via Orabona 4, 70126 Bari (Italy);

Email:cosimadamiana.calvano@uniba.it

Keywords: street art, pigments, mass spectrometry, binders, Py-GC/MS

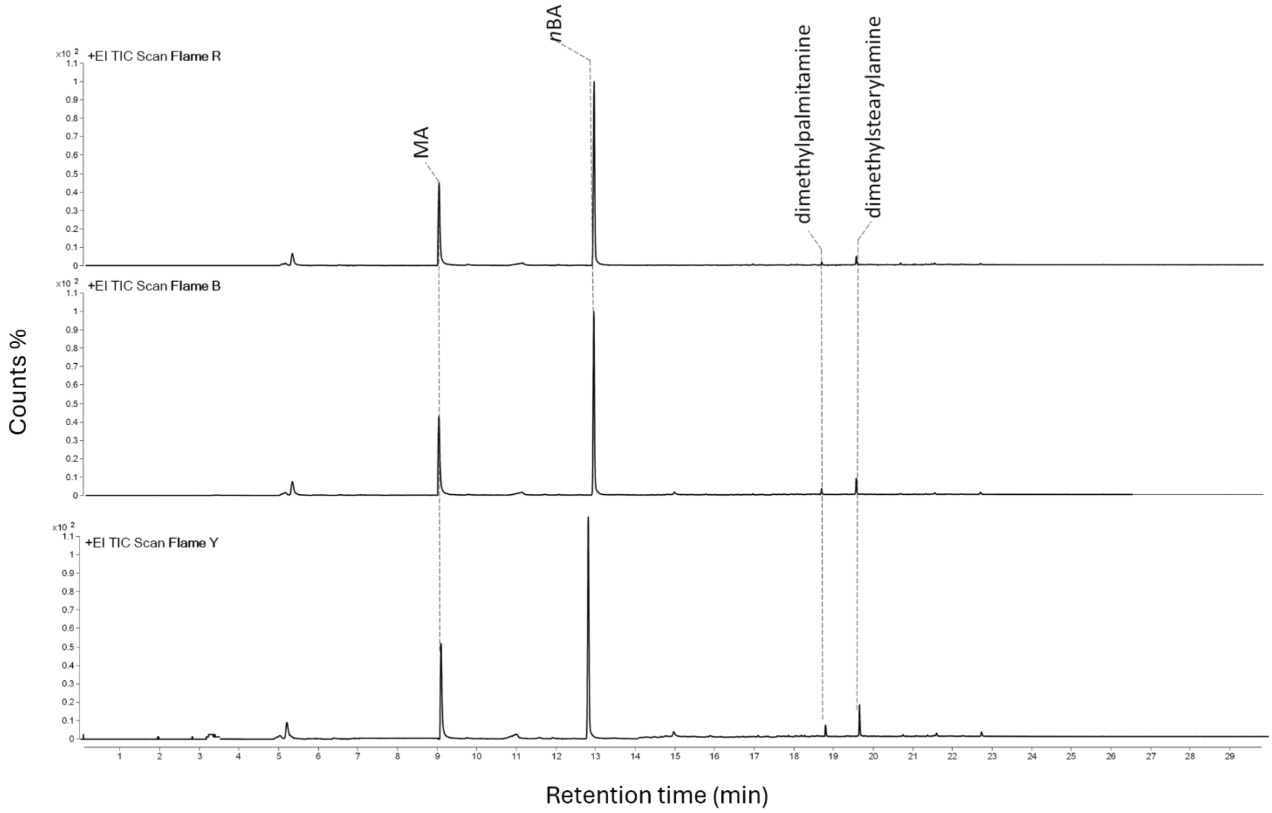

Figure S1. Pyrolysis-GC/MS chromatograms of three Molotow Flame Orange spray varnishes: (A) Magenta (Flame R), (B) Blue (Flame B), and (C) Yellow (Flame Y). Key identified components are labelled, including methyl acrylate (MA) and n-butyl acrylate (nBA) monomers characteristic of the acrylic binder system.

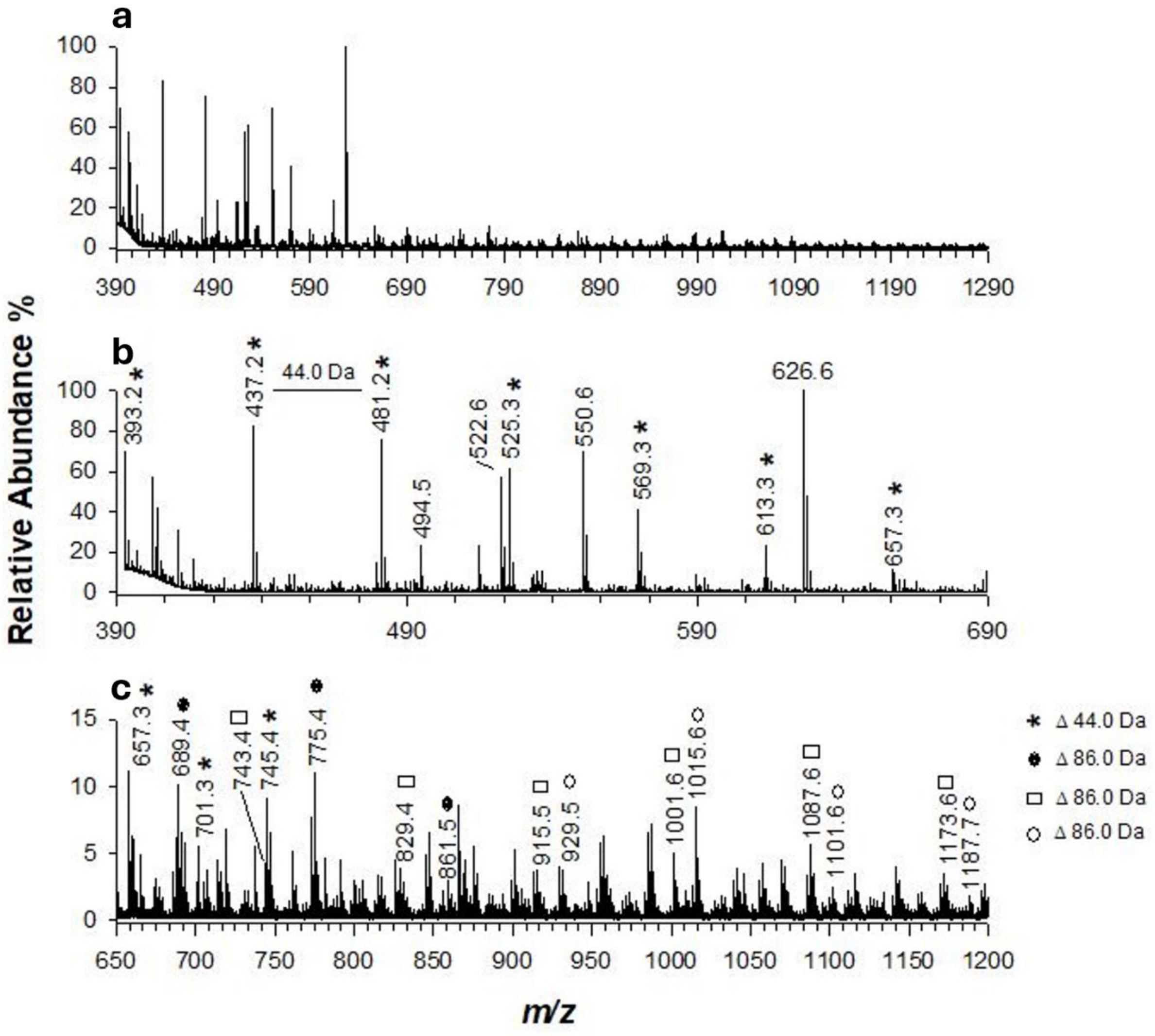

Figure S2. MALDI-ToF-MS(+) spectra of Molotow magenta (a) and enlargments showing PEG oligomers marked with * (b), and polymethyl acrylate oligomers (delta mass 86.0 Da, panel c).

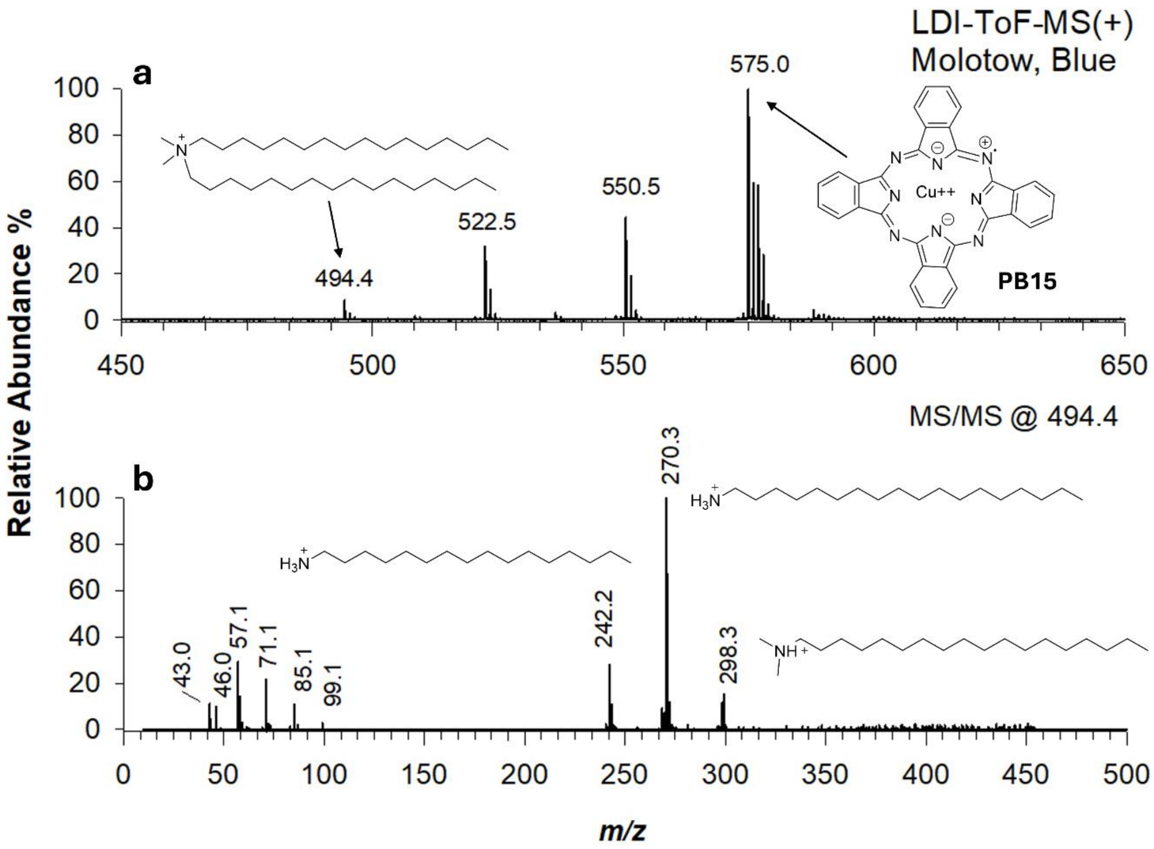

Figure S3. LDI-ToF-MS(+) spectrum of blue varnish Molotow (A) showing the presence of copper phthalocyanine at *m/z* 575.0. LDI-ToF-MS/MS(+) spectrum of the ammonium salt at *m/z* 494.4 (B). PB15 is represented as one of the possible resonance structures.

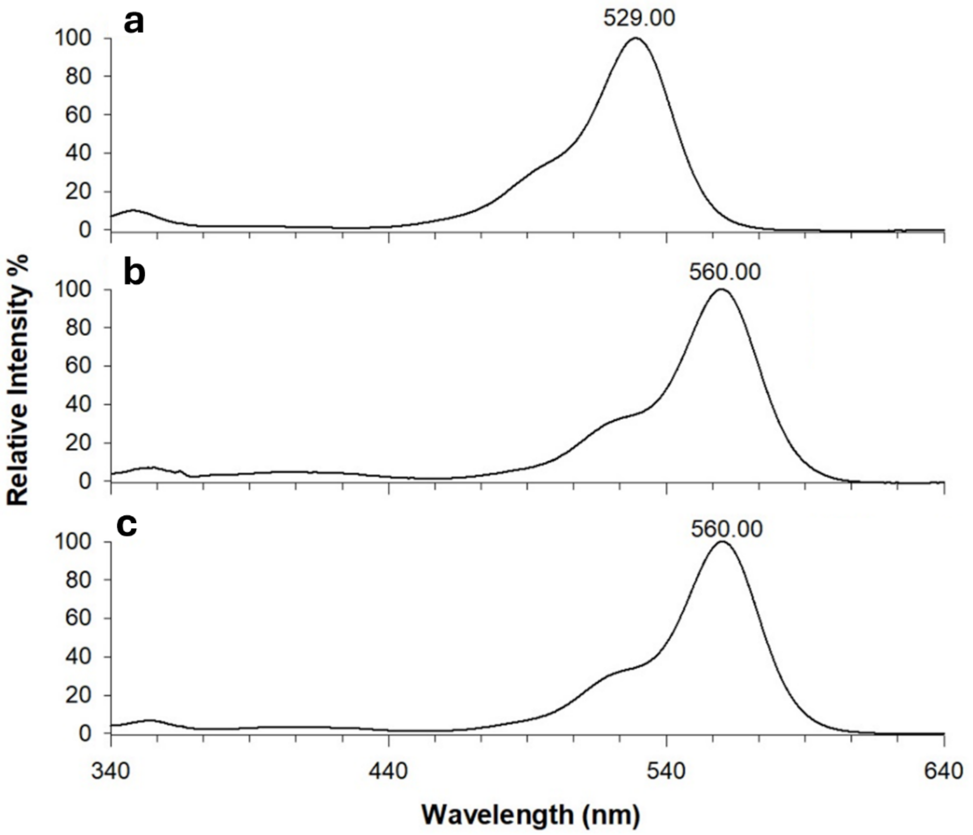

Figure S4. UV-visible absorption spectra of the three fluorescent colourants identified in Molotow pink varnish: (a) N-demethylated rhodamine 6G (t_R = 8.10 min), (b) N-methylated rhodamine B (t_R = 9.40 min), and (c) N-ethylated rhodamine B (t_R = 9.90 min).

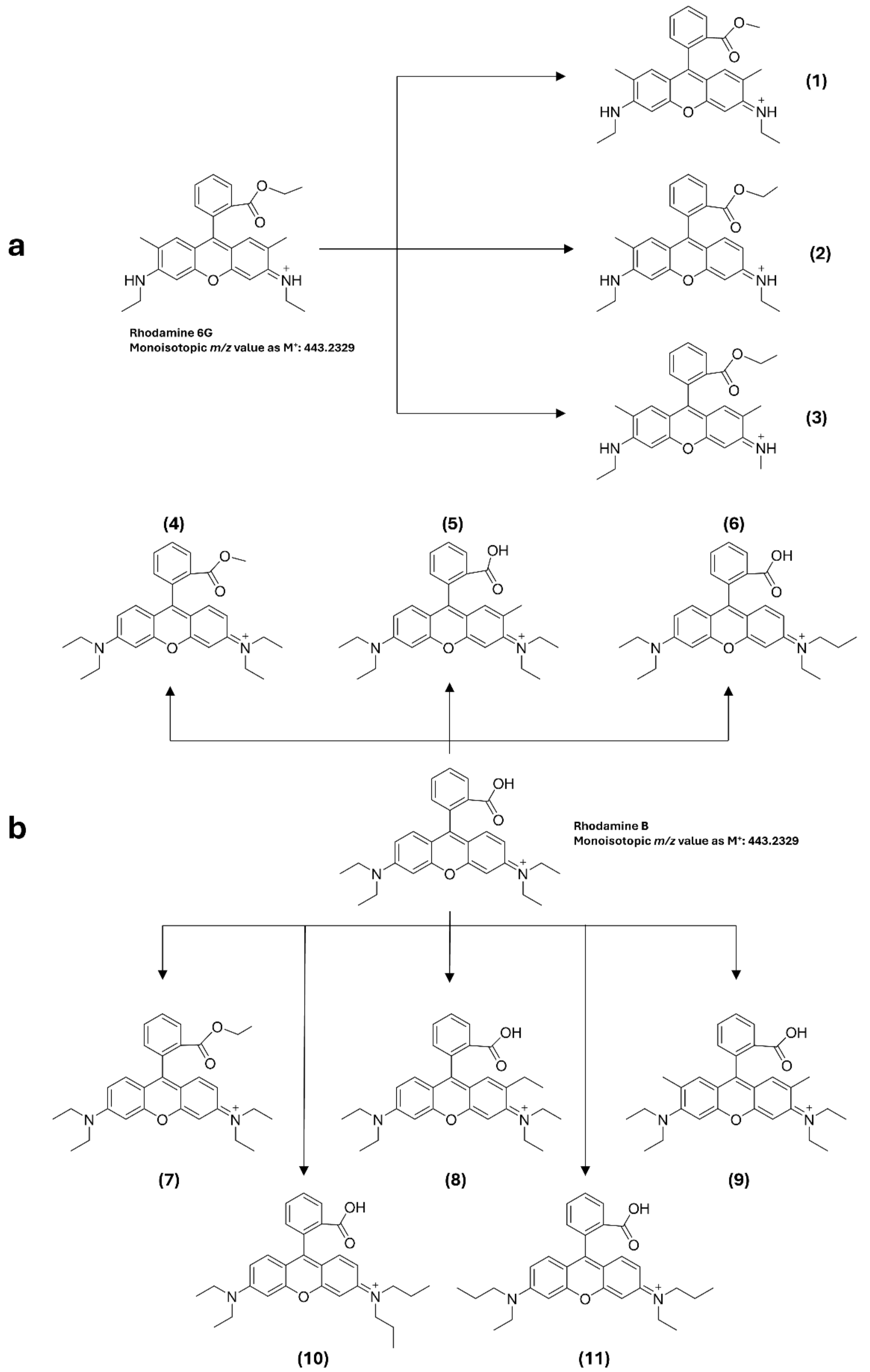

Figure S5. Chemical structures of (**a**) Rhodamine 6G and (**b**) Rhodamine B, with proposed derivative structures identified through high-resolution mass spectrometry. (a1-a3) Potential *N*-demethylated Rhodamine 6G derivatives (observed $[M]^+$ m/z 429.2173); (b4-b6) Potential *N*-methylated Rhodamine B derivatives (observed $[M]^+$ m/z 457.2486); (b7-b11) Potential *N,N*-dimethylated and N-ethylated Rhodamine B derivatives (observed $[M]^+$ m/z 471.2642). All mass measurements represent monoisotopic values.

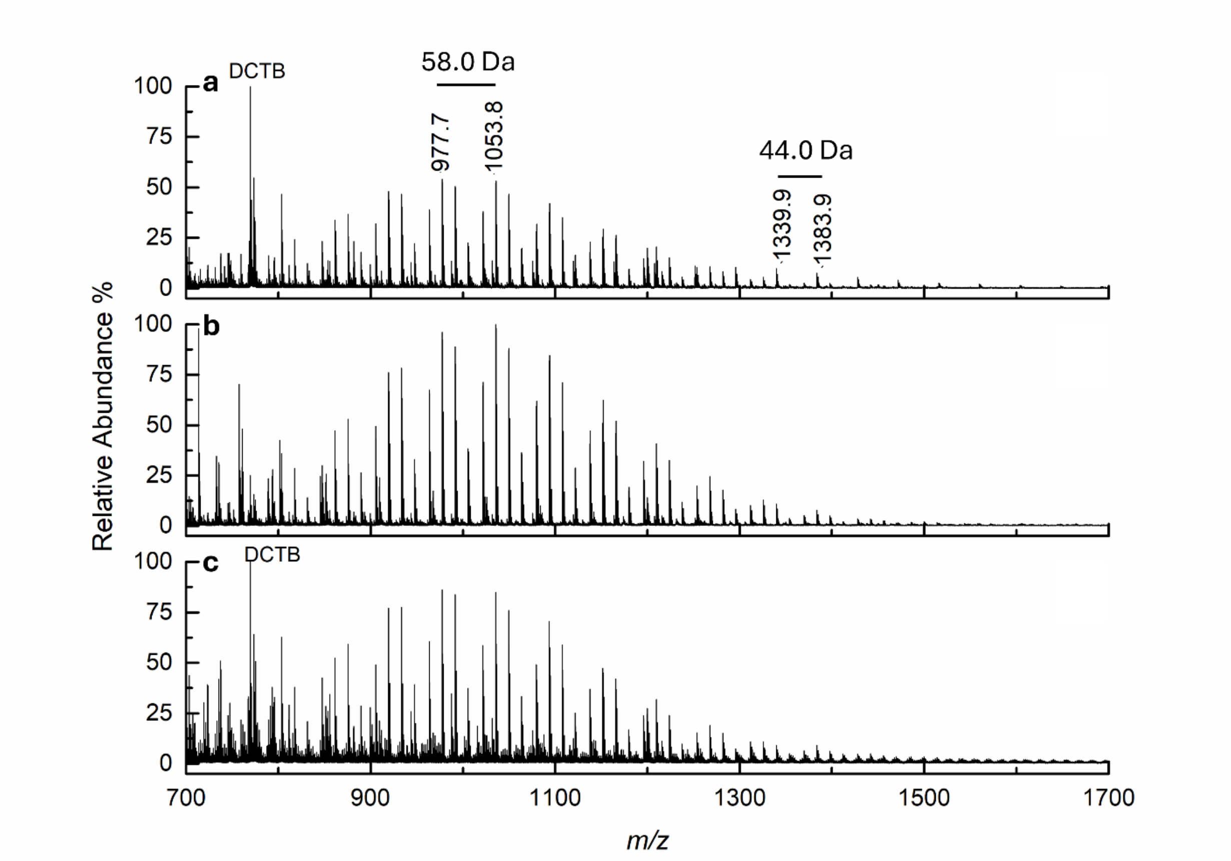

Figure S6. MALDI-ToF-MS spectra of the samples 1H, 2H (orange and yellow varnish, respectively panel a and b, from *Wikipedia Unusual Articles*), and 1M (Bordeaux varnish from *Metamorfosi,* panel c). Peaks labelled as DCTB are referred to MALDI matrix.

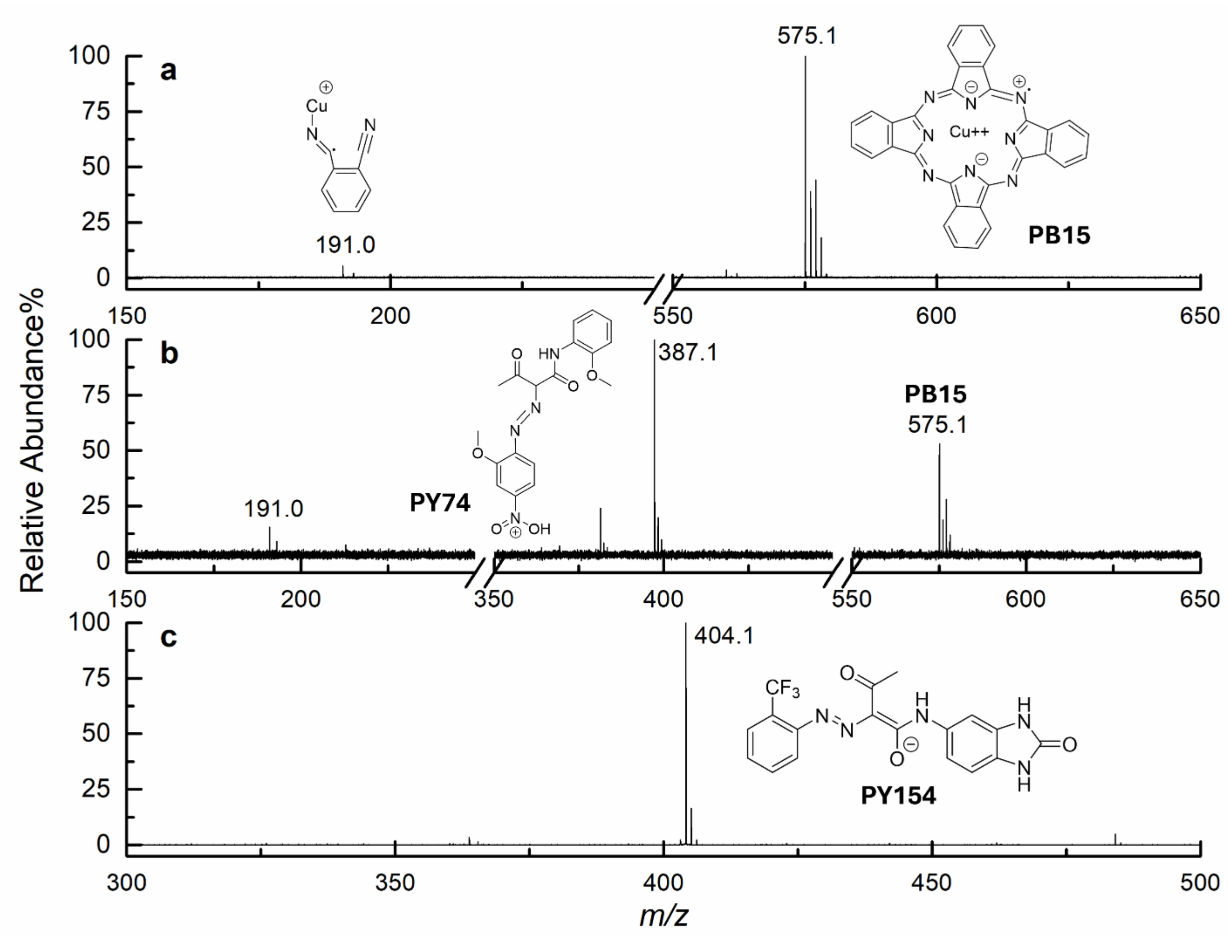

Figure S7. LDI-ToF-MS spectra of the samples 3H, 5H, and 2H (blue, violet and yellow varnish, respectively panel A, B and C, from *Wikipedia Unusual Articles*). PB15 is represented as one of the possible resonance structures.
